# Progressive embedding technique for large auricular cartilage-containing specimens in Mohs micrographic surgery

**DOI:** 10.1016/j.jdin.2026.04.012

**Published:** 2026-04-27

**Authors:** Thiago Rubim Bellott, Felipe Bochnia Cerci, Stanislav N. Tolkachjov

**Affiliations:** a Department of Dermatology, Fluminense Federal University, Niterói, Brazil; b Clínica Rubim Bellott, Santo Antônio de Pádua, Brazil; c Clínica Cepelle, Curitiba, Brazil; d Dermatology Service, Hospital Universitário Evangélico Mackenzie, Curitiba, Brazil; e Epiphany Dermatology, Dallas, Texas; f Texas A&M School of Medicine, Dallas, Texas; g Division of Dermatology, Baylor University Medical Center, Dallas, Texas; h Department of Dermatology, University of Texas at Southwestern, Dallas, Texas

**Keywords:** dermatologic surgery, frozen section, histologymethod, laboratory, Mohs micrographic surgery, pathology, processing, single section, skin cancer, quality assurance, quality control

## Challenge

The complex topography of the auricular region, with its alternating convex and concave surfaces, poses a considerable challenge in Mohs micrographic surgery.^1^ Large and irregular specimens, particularly those containing cartilaginous components, remain technically demanding during histologic processing. They often require division into multiple fragments, and cartilage can hinder appropriate embedding as well as prevent complete epidermal representation, among several other potential pitfalls.^2^ Furthermore, rapid freezing may result in uneven inclusion, incomplete epidermal display, and the formation of air bubbles beneath the tissue, affecting visualization of the deep margin in the sections.

## Solution

**Fig 1:**
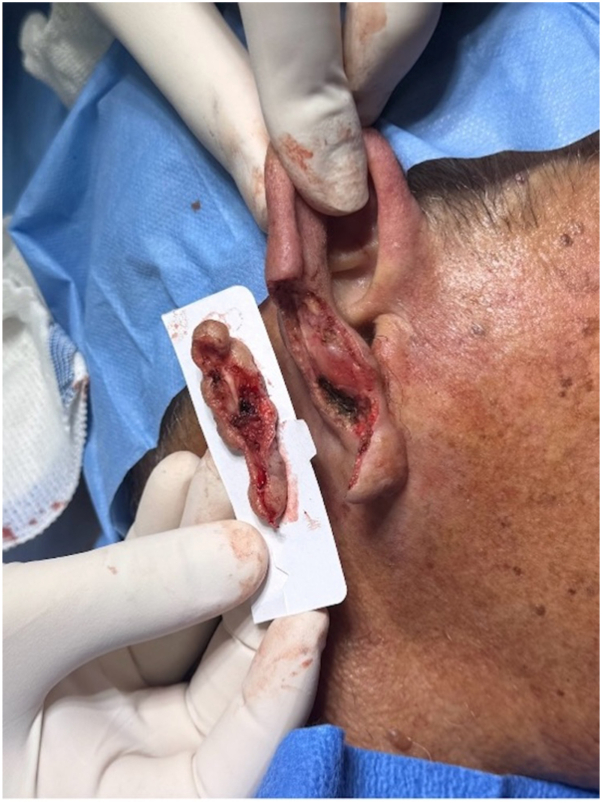
MMS specimen after removal of a squamous cell carcinoma from the ear, including cartilage, with a broad and irregular surface.

**Fig 2:**
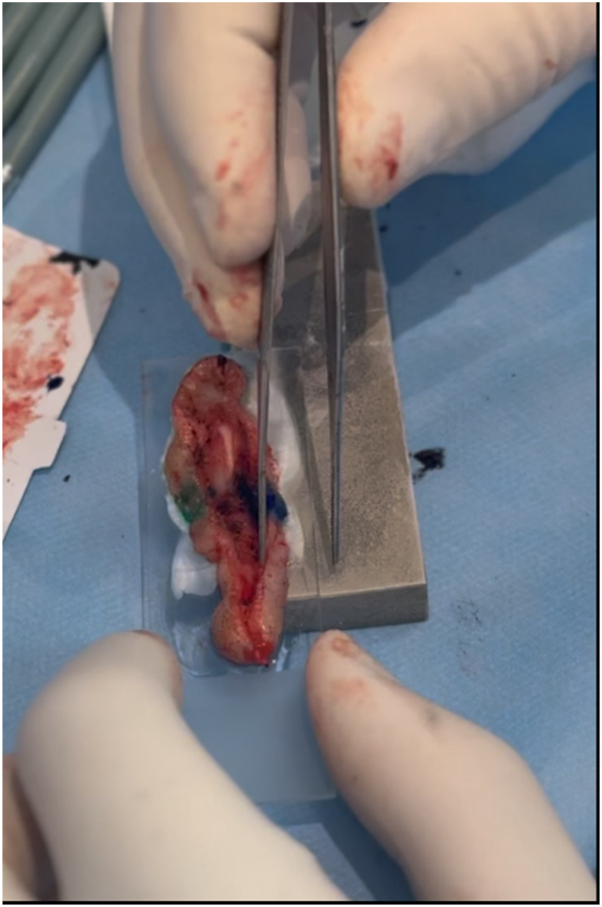
Specimen freezing. The whitening of the OCT medium indicates freezing of the specimen bottom, confirming proper adhesion to the slide surface.

We propose a simple and reproducible technique that enables embedding of these challenging specimens in a single section (Fig 1). Fresh tissue is placed directly onto a glass histology slide coated with a cryoembedding medium, positioned such that one end of the slide with the specimen rests on a pre-frozen aluminum plate. As the cryoembedding medium turns white, indicating freezing, the slide is advanced further along the aluminum block, allowing the remaining quadrants of the specimen to adhere and freeze in a controlled, sequential manner. Throughout the process, vertical pressure is gently applied, helping to adhere the frozen tissue to the slide (Fig 2) (Video, available on www.jaad.org). This approach allows more faithful representation of the entire specimen depth while ensuring that the epidermis lies flat on the lateral edges. For particularly large specimens, oversized glass slides and cryostat specimen holders (chucks) may be used instead of standard ones, avoiding unnecessary sectioning, thus reducing the risk of floaters or tumor dropout.

### Declaration of generative AI and AI-assisted technologies in the writing process

The authors used ChatGPT (OpenAI, San Francisco, CA) solely for language editing and refinement. All scientific content, interpretation, and conclusions were developed and approved by the authors.

## Conflicts of interest

Dr Tolkachjov is a speaker and investigator for Regeneron, Kerecis, Boehringer Ingelheim, and Castle Biosciences. Nascimento and Cerci have no relevant conflict of interest.

## Supplementary data

Video Demonstration of specimen embedding onto the glass slide by quadrants. Vertical pressure is applied, and the whitening of the glass surface together with tissue rigidity indicates adequate freezing and adhesion. Once a portion of the specimen is secured to the slide, the remaining quadrants are progressively adhered in the same manner.
